# A comprehensive approach to modeling maternal immune activation in rodents

**DOI:** 10.3389/fnins.2022.1071976

**Published:** 2022-12-16

**Authors:** Morgan C. Bucknor, Anand Gururajan, Russell C. Dale, Markus J. Hofer

**Affiliations:** ^1^School of Life and Environmental Sciences, Charles Perkins Centre, The University of Sydney, Sydney, NSW, Australia; ^2^The Brain and Mind Centre, The University of Sydney, Sydney, NSW, Australia; ^3^The Children’s Hospital at Westmead, Kids Neuroscience Centre, Faculty of Medicine and Health, The University of Sydney, Sydney, NSW, Australia; ^4^The Children’s Hospital at Westmead Clinical School, Faculty of Medicine and Health, The University of Sydney, Sydney, NSW, Australia

**Keywords:** maternal immune activation, prenatal stress, neurodevelopmental disease, inflammation, LPS, poly I:C

## Abstract

Prenatal brain development is a highly orchestrated process, making it a very vulnerable window to perturbations. Maternal stress and subsequent inflammation during pregnancy leads to a state referred to as, maternal immune activation (MIA). If persistent, MIA can pose as a significant risk factor for the manifestation of neurodevelopmental disorders (NDDs) such as autism spectrum disorder and schizophrenia. To further elucidate this association between MIA and NDD risk, rodent models have been used extensively across laboratories for many years. However, there are few uniform approaches for rodent MIA models which make not only comparisons between studies difficult, but some established approaches come with limitations that can affect experimental outcomes. Here, we provide researchers with a comprehensive review of common experimental variables and potential limitations that should be considered when designing an MIA study based in a rodent model. Experimental variables discussed include: innate immune stimulation using poly I:C and LPS, environmental gestational stress paradigms, rodent diet composition and sterilization, rodent strain, neonatal handling, and the inclusion of sex-specific MIA offspring analyses. We discuss how some aspects of these variables have potential to make a profound impact on MIA data interpretation and reproducibility.

## 1 Introduction

Epidemiological studies have been paramount for revealing the association between maternal health status during pregnancy and behavioral outcomes in offspring. What began this series of longitudinal investigations was interest in the long-lasting effects of maternal undernutrition on individuals born during the devastating Dutch Famine of 1944−1945 (Ravelli et al., 1986; Lumey, 1992; Lumey et al., 1993; Stein and Lumey, 2000; Painter et al., 2005). While this historical event remains tragic for many, it inadvertently developed a new approach to studying the etiology of disease. At present, it is well understood that a multitude of stressful events taking place during pregnancy can result in adverse health outcomes for the offspring later in life. The sources of maternal stress that lead to an increased risk of disease, however, are largely multifactorial. Well-studied human examples include instances of maternal inflammation due to infection or environmental factors such as, obesity, pollution, psychosocial stress or physical inactivity that can negatively impact normal fetal brain development and are associated with increased risk for neurodevelopmental diseases (NDD) − such as autism spectrum disorder (ASD), attention deficit hyperactivity disorder, Tourette’s syndrome and schizophrenia (SZ) (Meyer et al., 2008; Han et al., 2021a). The sequence of events that take place during neural development is highly regulated and involves extensive neuro-immune crosstalk. Cytokines for instance, are proteins of many trades. They play an important role in mediating immunity and inflammation in response to infection (Hofer and Campbell, 2016). During neural development, they assist with neurogenesis, neuronal migration, axon guidance, synapse formation and plasticity (Garay and McAllister, 2010). Therefore, an untimely dysregulation of their functional roles in response to maternal stress or infection can significantly alter the developmental trajectory of the fetal brain and influence the prospective mental health of the individual (Boulanger-Bertolus et al., 2018; Han et al., 2021b). This phenomenon is referred to as maternal immune activation (MIA) and reflects circulating levels of inflammatory markers such as cytokines that exceed the normal range during pregnancy (Boulanger-Bertolus et al., 2018). To provide context; under normal conditions, most cytokines are observed within the circulation at very low concentrations. However, levels can increase up to 1,000-fold in instances of infection (Garay and McAllister, 2010). Details regarding neuronal function, neurotransmitter systems, and immune alterations following MIA exposure, can be found here: (Bergdolt and Dunaevsky, 2019).

In the epidemiological realm, in-depth study of the maternal-fetal interface during pregnancy is limited, which is why the use of animal models to study such a complex phenomenon has served as such a powerful tool. A great deal of what we have come to understand has been due to exhaustive studies on rat and mouse models of MIA. The prevailing experimental approach relies on the simulation or induction of bacterial or viral infection during pregnancy using three main immunogenic agents, namely, the administration of lipopolysaccharide (LPS), polyinosinic:polycytidylic acid (Poly I:C) or influenza virus to the pregnant dam (Boksa, 2010). The most widely used immunogens are the Toll-like receptor mimetics LPS and Poly I:C, which as opposed to influenza induce a limited, well-defined innate immune response that allows for both the timing and intensity of MIA to be precisely controlled in an experimental setting (Bergdolt and Dunaevsky, 2019). Both agents have contributed toward a further understanding on how the timing and severity of infection during different windows of prenatal development can result in different pathological profiles in the offspring (Meyer et al., 2006; Garay et al., 2013; Giovanoli et al., 2015). However, significant heterogeneity has arisen from the use of LPS and Poly I:C models due to variation between batches and additional aspects relating to the type of mimetic used. The limitations of which has been addressed by many in recent years (Meyer and Feldon, 2012; Mian et al., 2013; Arsenault et al., 2014; Parusel et al., 2017; Kentner et al., 2019; Bauman and Van de Water, 2020).

Apart from the development of immunogen or pathogen-induced MIA models, other developmental models of prenatal stress have been established in the context of environmental gestational stress exposure. Although these paradigms have been explored primarily within the broader research area of early developmental programming and behavior; the leading experimental premise bears a striking semblance to the use of the MIA model worth discussing. Certainly, the classic infection-induced etiology of MIA is undoubtedly a critical player for increased NDD risk, but it is important to consider the role environmental exposures play during pregnancy as well; collectively termed the “exposome” (Han et al., 2021b). Environmental perturbations during pregnancy that result in either acute or chronic instances of inflammation in the human condition have been shown to include, dietary composition, frequency of exercise, adequate sleep, socioeconomic status, stress, exposure to pollutants, and the gut microbiome (Han et al., 2021a). The all-inclusive contribution of these factors has yet to be explored in an MIA rodent model but at present, there are reports from those who have explored the role of the maternal microbiome (Kim et al., 2017; Lammert et al., 2018), social stress or social isolation rearing (Vallée et al., 1997; Scarborough et al., 2021) and a maternal high-fat diet (Bordeleau et al., 2020) on the resulting molecular and/or behavioral alterations in developing offspring (the use of environmental gestational stress paradigms in rodents are extensively reviewed here: Brunton, 2013; Weinstock, 2017; Van den Bergh et al., 2020).

The sources of variation that stem from the laboratory MIA animal model is in fact, strikingly representative of the complexity taking place within the human condition. Those in the field are well-aware that several experimental parameters such as gestational window, the genetic background of mice, age of offspring for analysis, brain regions studied, and type of mimetic used, play a critical role in framing the evolving MIA narrative. These points have been addressed by several excellent reviews (Harvey and Boksa, 2012b; Bergdolt and Dunaevsky, 2019; Kentner et al., 2019; Bauman and Van de Water, 2020). However, to gain a more profound understanding of the sequence of events beginning *in utero* that ultimately lead to the manifestation of disease, it is critical to be aware of the many internal and external experimental variables that can confound MIA data interpretation. Here, we aim to provide research groups with a comprehensive experimental framework regarding variables that should be taken into consideration when designing an MIA animal study. These variables include confounding aspects of immunogen used, laboratory diet composition and sterilization processes, the implementation of environmental gestational stressors- such as, maternal obesity and psychological paradigms, the effects of rodent strain and sex-specificity in response to immune stimuli, the effects of neonatal handling and the importance of the inclusion of sex-specificity in offspring analyses. In addition, we, along with others, encourage the implementation of reporting guidelines to ultimately improve the models’ validity and reproducibility (Kentner et al., 2019).

## 2 Maternal manipulations

**FIGURE 1:**
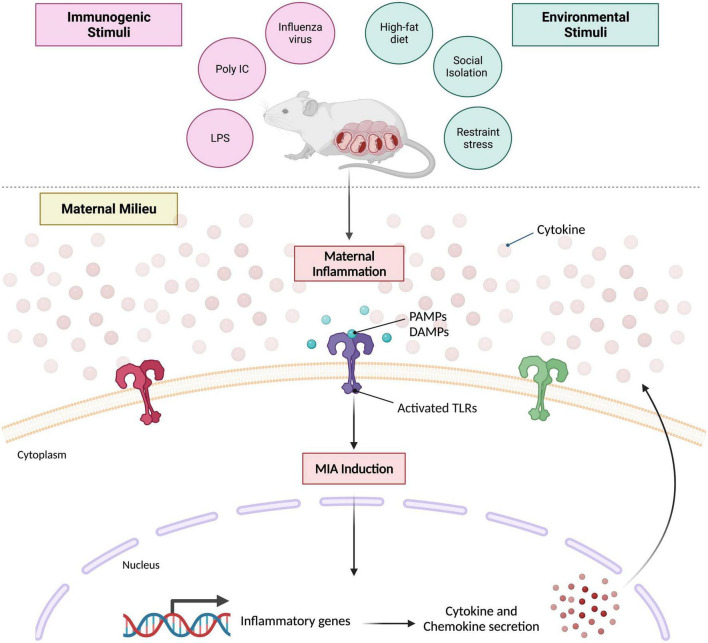
Prevailing mouse model of maternal immune activation. Maternal inflammation can be induced during various periods of gestation via immunogenic or environmental stimuli. Characteristic immunogens used that elicit an acute, innate immune response include influenza strain A or B, poly I:C (high- or low-molecular weight) and LPS. Environmental stimuli typically used include high-fat diet regimens, social stress paradigms or restraint stress. Despite the nature of the stimulus itself, both converge on the same molecular mechanism that evokes maternal immune activation. Pathogen associated molecular patterns (PAMPs) and/or damage associated molecular patterns (DAMPs) are generated which activate various toll-like receptors (TLRs) that promote the transcription of several inflammatory genes; ultimately leading to perpetual cytokine and chemokine secretion within the maternal milieu. Created with BioRender.com.

The most widely used and arguably, well-characterized manner of inducing MIA in rodent models is the administration of bacterial or viral mimetics, or various strains of live pathogens during different stages of pregnancy (Meyer, 2014; Weber-Stadlbauer and Meyer, 2019). There are plenty of advantages to examining prenatal exposure to live agents during pregnancy in a preclinical setting, but they do add a significant layer of complexity to the model system being studied. For one, they evoke the full spectrum of maternal immune responses – comprising of both innate and adaptive which can be challenging to monitor and measure appropriately. While most live pathogens remain restricted to the maternal circulation, placental transmission can take place in certain cases, such as rubella (Silasi et al., 2015). Their use in animal models of maternal infection and/or inflammation certainly helps establish and further solidify biological causality for epidemiological work that is tasked with determining the role of specific infectious agents (i.e., influenza) on disease-specific outcomes; such as schizophrenia for instance (Brown and Derkits, 2010). However, their use does come with significant biosafety precautions as well, that not all institutions have the capacity for. And, both the window and severity of live pathogens cannot be easily controlled *in vivo* as well. Importantly, a large part of why animal MIA investigations have gradually transitioned toward the use of mimetics is due to the commonality that lies between the induction of infectious and non-infectious agents and subsequent NDD risk – the maternal immune response (Figure 1). See other reviews here: (Meyer and Feldon, 2012; Estes and McAllister, 2016; Brown and Meyer, 2018). Mimetics in this context, refer to immunogenic agents that trigger the cytokine-associated inflammatory response in an acute manner. In addition, these agents lack the infectivity that most live pathogens possess.

### 2.1 Poly I:C model

An appealing alternative to inducing an exhaustive live infection while still inducing an acute maternal immune response is opting for the use of mimetics. The implementation of both bacterial (LPS) and viral mimetics (poly I:C) in MIA models have gained significant momentum for their ease of use, access, and well-defined nature of the resulting innate immune response. The viral mimetic responsible for eliciting the maternal anti-viral inflammatory response is a double stranded RNA (dsRNA) compound, poly I:C (Patterson, 2009). Poly I:C activates toll-like receptor (TLR)-3 in the same manner as dsRNA from live viruses, resulting in a signaling cascade that leads to the activation of transcription factor nuclear factor kappa B (NFκB) and subsequent transcription of genes that code for pro- and anti-inflammatory cytokines such as IL-1, IL-6, TNF, and type I interferons (e.g., IFN-α and IFN-β) (Boksa, 2010; Harvey and Boksa, 2012b; Meyer, 2014). The poly I:C model has successfully recapitulated key behavioral aspects of NDDs such as, schizophrenia (Meyer, 2014) and autism spectrum disorder (Carlezon et al., 2019); many of which have been reproduced (Patterson, 2009). Some reported examples include deficits in pre-pulse inhibition, social interaction, and novel object exploration; such observations have been extensively reviewed elsewhere (Patterson, 2009; Meyer and Feldon, 2012; Bergdolt and Dunaevsky, 2019). However, while behavioral outcomes have been well-characterized, other aspects of the model have come under some scrutiny. Initial reports of batch-to-batch poly I:C variation from the same vendor (Harvey and Boksa, 2012a) and later evidence demonstrating different molecular weights elicited contrasting cytokine responses in non-pregnant rats (Careaga et al., 2018), encouraged groups to seriously consider these sources of variation when using poly I:C to study MIA. These observations lead to an extension of these preliminary findings by Mueller et al. (2019), where they assessed different batches from the same vendor, low vs. high molecular weight, and contamination with LPS, a bacterial compound (described in section “2.2 LPS model” below) as an additional aspect of source variability in pregnant mice. Synthetic dsRNA analogs like poly I:C are hygroscopic substances and can be easily contaminated during product manufacturing or when reconstituted. Some vendors specify contaminating LPS content in their poly I:C products while others do not, which can lead to potential immunogenic inconsistencies (Mian et al., 2013) and emphasizes the importance of quality assessments.

However, the variability stemming from poly I:C molecular length is perhaps one of the most critical and compelling features of the dsRNA analog in terms of its downstream effects on maternal immune responses. The findings reported by Mueller et al. (2019) are strikingly consistent with Careaga et al. (2018) work done in nulliparous rats; demonstrating that administration of the high molecular weight (HMW) analog (1000−6000 nucleotides) to pregnant mice elicits a more robust host of maternal cytokine and chemokine responses in both plasma and placenta as opposed to the low molecular weight (LMW) analog (100−200 nucleotides). A plausible explanation for such a difference in immunogenicity lies in the efficiency of each analogs’ ability to activate TLR3. *In vitro* studies have identified LMW poly I:C to possess a high affinity for TLR3, while the HMW analog appears to selectively bind to retinoic acid inducible gene I (RIG-I) and melanoma differentiation-associated gene 5 (MDA5) receptors (Kato et al., 2008; Zhou et al., 2013).

Lastly, another important aspect of poly I:C to consider is its influence on pregnancy viability. Mueller et al. (2019) tested whether different batches of poly I:C could influence pregnancy outcomes in mice by assessing the rate of spontaneous abortions. They found batches released just a year apart from the same vendor (July 2016−2017) impacted abortion incidence. The more recent batch (July 2017) led to a higher incidence of spontaneous abortion as opposed to the older batch (July 2016). The authors’ note these observations could be partially due to more pronounced potency of the former compared to the latter. In addition to batch potency, gestational timing of administration was shown to play an important role. Of the two gestational windows tested (GD 9.5 and 12), the earlier window appeared to be more susceptible to abortion than the later window.

With these considerations in mind, it’s strongly encouraged that research groups attempt to address the following: (1) *Is it possible to conduct all poly I:C-based experiments using the same batch?* (2) *Is it most appropriate to opt for LMW or HMW poly I:C to administer during pregnancy?* (3) *Are details regarding LPS contamination disclosed from the vendor? If not, can this be easily tested in-house?* The route of administration (i.e., intravenously or intraperitoneally) on the other hand, is another point of contention surrounding the use of poly I:C; and been shown to result in noticeably different maternal cytokine profiles. The effects of which have been discussed elsewhere: (Meyer et al., 2006; Careaga et al., 2017; Mueller et al., 2019). Nonetheless, conducting a smaller, dose-response pilot study prior to much larger poly I:C experiments would be advised for determining optimal parameters specific to the MIA study.

### 2.2 LPS model

Another established immunogenic agent for modeling MIA in animal models at present, is the use of lipopolysaccharides (LPS) that mimics bacterial infection. LPS acts as an incredibly potent stimulator of the host innate immune system and its recognition is crucial for clearing infections by bacterial pathogens (Steimle et al., 2016). LPS is a component of the outer membrane of Gram-negative bacteria and a ligand for toll-like receptor (TLR)-4, resulting in the initiation of the innate immune response and subsequent inflammation (Depino, 2015). This well-characterized response consists of a robust increase in the expression of various cytokines and chemokines, such as IL-1β, IL-6, TNF, CXCL1, 2, 10 amongst others, along with increased corticosterone levels (Kentner et al., 2019). To our knowledge, it appears the narrative surrounding the utility of LPS for MIA animal models, has not received as much emphasis compared to the variable effects of poly I:C.

The structure of LPS consists of three core components: lipid A, core oligosaccharide and O-antigen repeats. Based on both size and structure, LPS is typically classified into three groups: smooth type LPS, which contains differing sizes of O-antigen repeats; rough type LPS, containing differing sizes of core oligosaccharides and no O-antigens; and free lipid A (Wang et al., 2010). Its structure ultimately varies amongst bacterial species, resulting in different bioactivity and degrees of immunogenicity within the host (Steimle et al., 2016; Parusel et al., 2017). When considering gestational LPS administration, it is important to take note of the potential consequences of commercial preparations on purity. Due to such structural variation, different methods have been developed to extract and purify LPS, such as extraction with phenol, water or ether (Wang et al., 2010). Each method leaves differing concentrations of contaminants (i.e., nucleic acids, lipoproteins, phospholipids, and proteins), which can amount to a mixed and non-specific innate immune response (Parusel et al., 2017). Standard LPS preparations are typically purified via phenol-extraction and contain both TLR-2 and TLR-4 activating agents (Parusel et al., 2017), which may be suitable if the aim is to mimic a more complete bacterial infection. A study done by Parusel et al. (2017) verified the signaling differences between LPS preparations by demonstrating an ultrapure preparation only activated TLR-4-dependent signaling, while the standard preparation activated both TLR-2 and TLR-4 in human embryonic kidney cells overexpressing murine TLR2. Primary TLR-2 ligands have been characterized as lipoproteins that are ubiquitously expressed amongst all species of bacteria and possess significant bioactivity (Hirschfeld et al., 2000; Oliveira-Nascimento et al., 2012). Depending on the nature of the proposed MIA experiment, one might opt for a more specific TLR-4 maternal immune response. In this case, the use of an “ultrapure” form of LPS that is almost entirely free of contaminants would be an appropriate alternative. Ultrapure preparations of LPS involve an additional phenol re-extraction protocol that effectively rids of contaminants (Hirschfeld et al., 2000; Tirsoaga et al., 2007), Accordingly, when interpreting maternal immune responses following LPS stimulation, it is wise to examine immune alterations carefully to ensure there is no over-interpretations. Unfortunately, details regarding the bacterial species of LPS used and its grade of purity are often not published, putting the reliability of experimental results into question and another important aspect that warrants further reporting. In the context of maternal LPS administration, we recommend considering the following queries: (1) *Do the scientific endpoints of the study support a non-specific or TLR-4-specific maternal immune response?* (2) *Which stereotype or species of LPS is most appropriate for the study?* (3) *How much is known regarding commonly used stereotypes of LPS on the robustness of the maternal immune response?*

In sum, many research laboratories have become increasingly aware of the various limitations surrounding the use of mimetics when designing an infection-based MIA model. Be that as it may, it can be argued that their limitations do not outweigh their many benefits. Besides, they have led to significant advancements in not only our knowledge concerning MIA and concomitant brain pathology and behavior in offspring; but the way in which laboratories are choosing to approach prospective MIA investigations. Investigators are more informed, and the practice of more rigorous reporting is slowly becoming more widespread. The abundance of literature pertaining to poly I:C and LPS-based models over the years have also opened the door for more exploration of the effects of other TLR-stimulating agents – Imiquimod and Resiquimod (Missig et al., 2020; Kwon et al., 2021); both single-stranded viral mimetics that selectively bind to either TLR7 or TLR8. In addition to these, some laboratories are shifting focus back to the effects of live pathogens during pregnancy as well, via administration of influenza strains and the parasite, *Toxoplasma gondii* (Jacobsen et al., 2021; Xu et al., 2021). Table 1 outlines various infectious and non-infectious agents used in recent investigations and resultant observations in MIA offspring.

**TABLE 1:** Immunogen-induced maternal immune activation (MIA) offspring alterations.

Compound	Species/strain	Mechanism of action	Gestational exposure	Key offspring observations	References
Poly IC (ds-RNA mimetic)	Mouse/C57BL/6	TLR3 agonist	GD 12.5	ISR pathway activation in M but not F offspring dependent on maternal IL-17a secretion ↓ global mRNA translation and protein synthesis in cortex of M offspring M fare worse than F offspring	Kalish et al., 2021
LPS (bacterial mimetic)	Rat/Wistar	TLR4 agonist	GD 9.5	M offspring analysis only: No change in anxiety-related behavior ↑ TNF, IL-6 mRNA levels in cortex in M offspring ↑ Plasma IFN-γ, TNF, IL-1 ↑ microglial activation ↑ ROS levels	Cieślik et al., 2020
Influenza virus	Mouse/C57BL/6	Wide-range innate immune response activating TLR3/TLR7/TLR8 and adaptive immune responses	GD 5.5	↑ vulnerability toward respiratory pathogens in early life (both sexes) ↑ vulnerability toward second-hit infections correlate with low birth weight (both sexes) ↓ alveolar macrophage capacity to clear respiratory pathogens (sexes pooled together)	Jacobsen et al., 2021
Imiquimod (ss-RNA mimetic)	Mouse/C57BL/6	Selective TLR7 agonist	GD 12.5, 14.5, 16.5	↓ F offspring born Conditional hyper-responsiveness (both sexes) ↓ anxiety-like behavior (primarily M offspring) Fragmented social behavior (primarily M offspring) ↓ Iba1 + microglia and ↑ ramified morphology (both sexes) Significant sex-specific transcriptional profiles in dorsal striatum	Missig et al., 2020
Resiquimod (ss-RNA mimetic)	Mouse/C57BL/6	TLR7/TLR8 agonist	GD 12.5	↑ IL-6, TNF, Ccl2, Ccl5 fetal brain mRNA levels ↑ fetal brain microglial marker expression via Aif1 (Iba1) expression	Kwon et al., 2021
Toxoplasma gondii (parasite)	Mouse/C57BL/6	TLR11/TLR12 agonist	GD 14.5	M offspring analysis only: Autism-relevant behaviors Aberrant brain microstructure Peripheral pro-inflammatory T-cell profile ↑ IL-6 in astrocytes Adoptive transfer of T_reg_ cells significantly reversed MIA-phenotype	Xu et al., 2021

TLR, toll like receptor; GD, gestational day; ISR, integrated stress response; IL-6, interleukin-6; IL-17a, interleukin 17a; IL-1, interleukin 1; IFN-γ, interferon gamma; TNF, tumor necrosis factor; Ccl2/5, chemokine (C-C motif) ligand 2 and 5; T_reg_, regulatory T cell; M, male; F, female; ROS, reactive oxygen species.

### 2.3 Psychological induction of MIA

Thus far, the discussion has centered on the infection-induced etiology of MIA in animal models and the many confounding variables to take into consideration when designing a maternal infection-based MIA study. Here, we will switch the focus of discussion to what comprises a non-infection-based model of MIA; which includes modeling various sources of maternal stress originating from the environment or the “exposome” (Table 2). We will discuss established psychological stress paradigms, as well as maternal obesity in Section “2.4 Maternal obesity induction and MIA.” Both stress and obesity readily activate the maternal immune system and represent maternal factors that are becoming increasingly relevant in the human condition and predisposition of NDDs.

**TABLE 2:** Environmental-induced MIA offspring alterations.

Paradigm	Species/strain	Gestational exposure	Key offspring observations	References
High-fat diet	Mouse/C57BL/6	4 weeks prior to gestation, throughout gestation and nurturing till weaning	Exaggerated inflammatory response to peripheral LPS in HFD-exposed M and F offspring Microglia morphology alterations ↑ microglia interactions with astrocytes in hippocampus in M offspring ↓ microglia-associated extracellular space pockets in hippocampus in both M and F ↓ TGFb1, Tmem119, Trem2, Cx3cr1 mRNA levels in hippocampus significantly in M offspring	Bordeleau et al., 2020
Chronic social instability (CSI)	Mouse/CD1	2x/week for 7 weeks prior to gestation	↑ anxiety and social deficits predominantly inherited by F offspring across 3 generations Complete maternal and paternal transmission of CSI-related deficits can be transmitted to F1 offspring Differential transmission between maternal and paternal line in F2 and F3 generations	Saavedra-Rodríguez and Feig, 2013
Social isolation rearing	Mouse/C57BL6/N	10-weeks prior and throughout gestation (GD0-20 ± 1)	M and F offspring ↑ anxiety-like behavior, cognitive impairments, and alterations to amygdalar transcriptome M offspring ↑ robust cognitive impairments in spatial recognition and temporal order memory M offspring ↑ vulnerable to deficits	Scarborough et al., 2021
Electric shock	Rat/Sprague-Dawley	Every other day for 10, 90-min sessions during gestation	↑ secretion of HPA hormones (plasma CORT and ACTH)	Takahashi and Kalin, 1991
Restraint stress	Mouse/C57BL/6 WT C57BL/6 GF CCL2^–/–^ KO	GD10-16	WT: ↑ brain IL-6 gene expression, ↑ Ccl2 protein levels, ↓ brain TLR4 gene expression GF: no fetal brain inflammation KO: no change in brain IL-6 gene expression levels, ↓ TLR4 brain gene expression, no sociability, or anxiety-related deficits	Chen et al., 2020
Noise at 95dB	Rat/Wistar	Third trimester of gestation 1, 2, and 4 h/day for 7 days	Impaired hippocampal-dependent learning and memory, lower LTP, ↑ corticosterone	Barzegar et al., 2015
Food or water deprivation	Mouse/Rockland-Swiss albino	GD13-18 3 × 30 min sessions/day	No differences observed in litter size or pup mortality. Pup weight did not significantly differ between additional groups.	Kinsley and Svare, 1986
Social defeat by resident lactating female	Rat/Sprague-Dawley	GD16-20; 10 min sessions	↓ female birth weight Exaggerated ACTH and CORT responses to IL-1β immune challenge and restraint stress in both M and F	Brunton and Russell, 2010
Repeated variable prenatal stress regime	Rat/Sprague-Dawley	GD14-22/23 1−3 sessions/day	↑ locomotor activity, stereotypic behaviors ↑ sensitivity to amphetamine ↑ aggressive behavior toward adult and juvenile rats of the same gender ↑ delay-dependent deficits in recognition memory	Wilson and Terry, 2013

LPS, lipopolysaccharide; HFD, high fat diet; GD, gestational day; TLR, toll like receptor; TGFb1, transforming growth factor beta 1; Tmem119, transmembrane protein 119; Trem2, triggering receptor expressed on myeloid cells 2; Cx3cr1, CX3C chemokine receptor 1; IL-6, interleukin-6; Ccl2, chemokine (C-C motif) ligand 2; IL-1β, interleukin 1 beta; HPA, hypothalamic–pituitary–adrenal; CORT, corticosterone; ACTH, adrenocorticotrophic hormone; WT, wild type; GF, germ free; KO, knockout; LTP, long-term potentiation; M, male; F, female.

There are multiple social stress paradigms that have been designed to effectively mimic maternal social stress in rodent models. However, these paradigms have largely been implemented in isolation of additional gestational stressors (i.e., infection) and warrants further study with the aim to model an extremely multifactorial phenomenon, which would reflect the situation in humans more accurately. Evidence is emerging that maternal stress during pregnancy manifests as systemic, chronic inflammation (SCI) which has become a significant risk factor for the development of NDDs (Kim et al., 2019; Han et al., 2021a,b). The various triggers of maternal stress and subsequent SCI, include maternal lifestyle and environmental exposures, collectively termed the “exposome.” The exposome refers to − chronic infections, physical inactivity, visceral obesity, microbiome dysbiosis, social isolation, psychological stress and exposure to environmental hazards such as, air pollutants or tobacco smoking (Furman et al., 2019). Many instances of acute infections can in fact be accompanied by additional psychosocial or environmental factors that may prevent the speedy resolution of the initial acute inflammatory response and instead, perpetuate a state of low-grade SCI that ultimately causes significant collateral damage to peripheral organs and tissue over time (Furman et al., 2019; Han et al., 2021a). This reiterates the notion that MIA is very much a consequence of complex interactions between heterogeneous maternal inflammatory states over the course of pregnancy and it is therefore critical to incorporate such heterogeneity into preclinical animal models moving forward.

To date, modeling social and/or any other form of maternal environmental stress during gestation has been done almost exclusively in rats (Zuena et al., 2008; Brunton and Russell, 2010; Schroeder et al., 2013; Zhao et al., 2013; Hove et al., 2014; Palacios-García et al., 2015; Zohar et al., 2016). Needless to say, modeling an alternative form of maternal stress that is relevant to the human experience in rodents does present somewhat of a challenge. Nonetheless, successful attempts have been made in this area over the years and various paradigms have emerged, including − repeated restraint exposure (Henry et al., 1994; Zuena et al., 2008; Brunton and Russell, 2010; Schroeder et al., 2013; Zhao et al., 2013; Palacios-García et al., 2015; Zohar et al., 2016), noise at 95 dB (Barzegar et al., 2015), housing and/or social defeat with a lactating rat (Henry et al., 1994; Bosch et al., 2007; Brunton and Russell, 2010; Barzegar et al., 2015), electric shock (Takahashi and Kalin, 1991), food or water deprivation, reversal of light-dark cycle, cage tilting (Pryce and Fuchs, 2017) and a repeated variable prenatal stress regime (this entails the introduction of a number of random stressors on an unpredictable schedule) (Markham et al., 2010; Schulz et al., 2011; Wilson and Terry, 2013; Sickmann et al., 2015). At the cellular level, these environmental stress paradigms trigger elevations in various pro-inflammatory cytokines (i.e., IL-1β, TNF, and IL-6), activation of the transcription factor nuclear factor-κB and other proteins in the CNS (García-Bueno et al., 2008). Psychosocial paradigms such as chronic restraint, social isolation and repeated social defeat appear to result in increased microglial activity primarily in the hippocampus (Calcia et al., 2016). Further discussion concerning proposed neuroinflammatory and neuroendocrinological mechanisms following physical-psychological paradigms in rodents can be found here: (García-Bueno et al., 2008; Calcia et al., 2016; Liu et al., 2017; Picard et al., 2021). Restraint stress is particularly popular and a relatively easy intervention to use in rodents. It serves as a prenatal stressor in a physical sense primarily for the pregnant dam and secondarily, exerts detrimental effects to the developing offspring. This intervention typically involves the placement of the dam into an apparatus that restricts the degree of movement and consists of an opening at the top allowing for adequate air exchange. This can be applied up to thrice daily for 45 min each time (Hove et al., 2014) or once daily for 60 min (Fujita et al., 2010), and can be applied in addition to another gestational stressor. Another well-established stress regime includes the resident-intruder paradigm. This paradigm involves social stress in a primal sense and is measured as “social defeat” exposure. This entails the use of social conflict between an “intruder” animal that is transferred into the home cage of the “resident” for a specified period. This paradigm is typically carried out in male rodents, as males possess more of a tendency to exhibit aggressive behavior opposed to females (Brunton, 2013), but this paradigm has been adapted for use in pregnant female rats. Females can exhibit aggressive behavior during the first half of the lactation period in response to an intruder approaching their nest to protect their pups (Brunton, 2013). This maternal response was adapted by Brunton and Russell (2010), where pregnant “intruder” rats were placed in a cage with an unfamiliar lactating “resident” for 10 min/day on gestation days 16−20 of pregnancy. They observed heightened hypothalamic-pituitary-adrenal (HPA) axis responses in both male and female offspring of socially stressed mothers, with male offspring displaying significantly increased anxiety-like behavior. Further, mineralocorticoid receptor mRNA levels were reduced in the hippocampi of prenatally stressed male and female offspring, and both corticotropin-releasing hormone mRNA and glucocorticoid receptor mRNA expression in the central amygdala was found to be increased compared to control offspring. These data highlight the effects of a primal, maternal social stressor on the developing neuroendocrine system in both male and female offspring, and importantly, how this prenatal stress manifests in a sex-dependent manner. However, while these maternal stress regimens described appear to impose lasting alterations on fetal neurodevelopment and other associated-behavioral consequences in offspring, the limitation to these paradigms is their largely physical nature and failure to capture a more clinically relevant social component.

Two prevailing methods of imposing social stress on laboratory housed rodents that are arguably most related to aspects of human social behavior, are the social isolation and social instability stress paradigm (Table 3). One study modeled maternal depression in mice by incorporating social isolation both before and during gestation; concomitantly with antidepressant use (fluoxetine) (Scarborough et al., 2021). The social isolation paradigm *per se*, is not what makes this study unique. In fact, the use of a social isolation or separation-based depression model has been used frequently in rodents (Martin and Brown, 2010; Kumari et al., 2016; Huang et al., 2017; Mumtaz et al., 2018), albeit not during gestation. In brief, they identified sex-specific alterations in the offspring born to socially isolated mothers, which were rescued by maternal fluoxetine treatment in male, but not female offspring. Specifically, increased anxiety-like behavior was observed in both male and female offspring, whereas cognitive deficits were observed only in male offspring. Alterations to the transcriptome of the amygdala in both male and female offspring were analyzed, revealing profound male-specific effects. Most genes associated with memory were downregulated in male pups born to socially isolated mothers without antidepressant treatment. The administration of fluoxetine during pregnancy appeared to largely reverse this transcriptional signature – showing most of those genes to be upregulated as a consequence of maternal antidepressant use.

**TABLE 3:** Rodent social stress paradigms.

Paradigm	Species/strain	Stress regimen		References
		**Starting age**	**Length of exposure**	
Social isolation rearing (SIR)	Mouse/C57BL6/N	PnD 21	7 weeks	Scarborough et al., 2021
Separation-based model of depression	Mouse/C57BL/6J	Group 1: PnD 56 Group 2: PnD 161	Group 1: 16 weeks Group 2: 1 week	Martin and Brown, 2010
Social isolation housing	Mouse/ ddN	**Exp. 1** Group 1: PnD 35 Group 2: PnD 49 **Exp. 2** Group 1: PnD56	**Exp. 1** Group 1: 3 weeks Group 2: 1 week **Exp. 2** Group 1: 3 weeks	Orikasa et al., 2015
Social isolation housing	Rat/Sprague-Dawley	**Juvenile social isolation** PnD 21 **Gestational social isolation** PnD 51	30 days 20 days	Pisu et al., 2017
Social isolation housing	Rat/Sprague-Dawley	PnD 30	30 days	Pisu et al., 2016
Social isolation housing	Rat/Sprague-Dawley	PnD 25−30	30 days	Pisu et al., 2013
Social isolation housing	Mouse/swiss albino strain	PnD 56	8 weeks	Kumari et al., 2016
Social separation	Rat/Wistar	Group 1: PnD 75 Group 2: PnD 75 Group 3: PnD 75	1 week (GD 1−7) 1 week (GD 8−14) 1 week (GD 15−21)	Soliani et al., 2017
Social instability stress (SIS)	Mouse/C57BL/6J	PnD 56	Every 3 days for 7 weeks	Yohn et al., 2019
Chronic social instability	Mouse/CD1	PnD 27	2x/week for 7 weeks	Saavedra-Rodríguez and Feig, 2013
Chronic social instability	Rat/Sprague Dawley	PnD 60−70	Every 2−3 days for 4 weeks	Pittet et al., 2017

PnD, postnatal day; GD, gestational day.

The social instability stress (SIS) paradigm on the other hand, takes advantage of the hierarchical nature of a rodent, unisexual group-housing environment. While the specifics of the protocol can vary across laboratories (i.e., cage density, duration of protocol, frequency, and timing of cage changes), the fundamental component entails frequent re-shuffling of cage group composition several times per week. This paradigm introduces unpredictable social stress by consistently introducing animals to unfamiliar same-sex subjects into a new social environment and thereby limiting the ability to form stable social dynamics and/or hierarchies within the cage (more detailed discussion can be found here: McCormick and Green, 2013; Scharf et al., 2013; Koert et al., 2021). Many SIS paradigms have not been previously implemented during gestation apart from one study that analyzed the transgenerational effects on offspring (Saavedra-Rodríguez and Feig, 2013). Following cross-fostering experiments on SIS offspring, Saavedra-Rodríguez and Feig (2013) demonstrated transgenerational transmission of SIS deficits was in fact genetic and not due to significant changes in maternal care. In addition, the SIS phenotype in offspring was found to be exaggerated when both parents had been exposed to SIS stress. These investigations present novel approaches to modeling social stress in rodents and would be worthwhile additions to the “classic” MIA model. In particular, how social isolation housing or SIS works in synergy with other commonly used MIA stimuli (i.e., Poly I:C, LPS, and influenza) would be more representative of the multifactorial nature of MIA in humans and crucial for improving these models’ translational value. Moving forward, we urge investigators to keep the following considerations in mind (1) *Does the preferred social stress paradigm need to be optimized for use in females?* (2) *How important is timing* − *will the protocol be most appropriate to implement prior to or during gestation?* (3) *Are the scientific endpoints of the study sensitive to whether or not the psychological stress is more physical or social in nature?* (4) *Are there biochemical assays or behavioral tests that can reliably measure psychosocial stress in the experimental animals?*

### 2.4 Maternal obesity induction and MIA

The prevalence of maternal obesity [classically defined as a body mass index (BMI) of >30.0 in humans] is progressively increasing and the ramifications to the developing offspring is an incredibly important aspect of studying MIA. In the United States, the CDC reported an increase in pre-pregnancy obesity from 26.1% in 2016 to 29.0% in 2019 – an increase of 11% (Driscoll and Gregory, 2020). This trend was also observed amongst women across all maternal ages, race, and educational levels (Driscoll and Gregory, 2020). Obesity in and of itself, presents a host of adverse metabolic defects and frequently coexists with inflammation (Lee et al., 2013; Murabayashi et al., 2013; Ellsworth et al., 2018). Adipose tissue was long believed to be biological inert, however it is now recognized as an active endocrine tissue that mediates immunity and inflammation (Galic et al., 2010). Obese adipose tissue is resident to many activated macrophages which eventually leads to the overproduction of several pro-inflammatory cytokines termed adipokines, such as, IL-6 and TNF, of which are implicated in the development of metabolic dysfunction (Galic et al., 2010; Lee et al., 2013). Maternal obesity during pregnancy is especially problematic and represents one of the many lifestyle factors presented earlier in the discussion known to contribute toward SCI and subsequently, MIA. Moreover, epidemiological studies have revealed significant associations between maternal obesity and the onset of ASD and ADHD in offspring (Han et al., 2021b). Several diet-induced obesity studies have been conducted in rodent models to better dissect the many short- and long-term developmental programming effects of maternal obesity on various metabolic and cognitive parameters in the offspring (Samuelsson et al., 2008; Hartil et al., 2009; Kirk et al., 2009; White et al., 2009; Sasson et al., 2015; Bordeleau et al., 2020; Lippert et al., 2020). Yet, the induction of maternal obesity in the context of an MIA investigation is seldomly done (in rodents) and warrants further study. To date, only one recent mouse study sought to directly illustrate the induction of MIA via a maternal high-fat diet and alterations to the offspring’s microglial gene expression profile and subsequent behavior (Bordeleau et al., 2020). However, pregnant dams were not characterized as “obese” or exhibited metabolic changes that would be consistent with obesity (i.e., hyperglycemia, hyperinsulinemia, and/or dyslipidemia). Therefore, the authors note that the changes observed in the offspring were due to the maternal immune system’s response to a diet enriched in fat as opposed to obesity-induced metabolic dysregulation. Questions have also been raised in recent years concerning the standardization of rodent diets used to induce obesity and whether or not these models are accurate representations of human obesity (Wankhade et al., 2016; Speakman, 2019). Thus, concerns remain about the best way to both model and measure obesity in rodents (as they are the most widely used model organism); and ultimately reproduce metabolic data between research groups. One reasonable explanation for why there tends to be such large variation in metabolic data using rodent models is partially due to the fact that mice from different institutions utilize food for energy at different rates, making it difficult to determine what metabolic state can be considered “normal” (Corrigan et al., 2020). Obesity being essentially a chronic state of positive energy balance, shifts the metabolism from efficient energy utilization to predominantly storage. Therefore, energy expenditure can be a very useful measure when it comes to characterizing and potentially predicting the onset of obesity in a well-controlled laboratory study. Indirect calorimetry is an example of a popular method that has emerged to measure energy expenditure by measuring oxygen consumption and carbon dioxide production in real time (Lam and Ravussin, 2017).

As to what constitutes a “high-fat” diet in a laboratory setting is another fair point of contention. The standard fat content present in the rodent diet is approximately 10% which can come from various sources such as lard, corn oil, safflower oil, coconut oil, soybean oil or menhaden oil (Wankhade et al., 2016; Weiskirchen et al., 2020). Typically, high-fat diet studies conducted in rodents consist of diets containing either 45% or 60% fat and high sucrose content. Diets containing 45% fat are often referred to as a “Western” diet in the literature, whereas 60% high fat diets are often termed “High Fat” (Wilson et al., 2007; Ballal et al., 2010; Bruder-Nascimento et al., 2017; Chaix et al., 2021). Many laboratories opt for a 60% high-fat diet as mice become obese more rapidly, which thereby reduces cage costs and is more convenient (Speakman, 2019). However, a 60% high fat diet is an extreme example of diet-induced obesity and does not accurately reflect the typical Western dietary composition in humans (Hintze et al., 2012). The issue surrounding the appropriate translation of high-fat diet studies in rodents has been an ongoing concern for some time and was discussed at length by an editorial published by Speakman (2019). According to Speakman, the stereotypical American or European diet is estimated to contain roughly 36−40% total calories from fat, while the upper end of the spectrum may contain up to 50−60% of calories from fat. Yet, this is not to be confused with the ketogenic diet which entails up to 80−90% of caloric intake be from various fat sources (Allen et al., 2014; Cabrera-Mulero et al., 2019). What is important to note in the diet-induced obesity area of research in rodents is the comparison between an animal fed a standard diet consisting of 10% fat vs. an animal fed a high-fat diet of 60%. Certainly, this analysis can be considered largely skewed and not an accurate reflection of the human condition. Therefore, the “Western” high fat diet (45%) may be considered more well-suited for use in the context of translation and may result in the improved validity of analysis between cohorts of animals.

When it comes to comparing measures of obesity across mouse models, both intrinsic and strain variations have been shown to exert confounding effects, making the validity and reproducibility of metabolic data collected difficult to compare between groups. An investigation using more than 30,000 male C57BL/6J mice illustrated that large phenotypic variation exists in mice energy expenditure, despite a virtually identical experimental design across four institutions around the world (Corrigan et al., 2020). The primary variables that appeared to be responsible for this variation included: mouse body composition, ambient room temperature and institutional location of the study. The Corrigan group recommend reporting these additional parameters in further metabolism studies for better transparency and reproducibility between groups. The widely used C57BL/6J is also typically considered an obesity-prone sub-strain, as they exhibit an array of obese biomarkers (i.e., marked weight gain, hyperinsulinemia, lipid accumulation in the liver and adipose tissue, and amongst other parameters), while the A/J strain appears to be obesity-resistant (Surwit et al., 1995; Nilsson et al., 2012; Li et al., 2020). An additional report studying strain-specific differences found BALB/c mice to be less-susceptible to high-fat diet induced obesity (Montgomery et al., 2013). Further strain-specific responses to dietary obesity has been characterized in earlier work (West et al., 1992, 1995). Sex-specificity is another critical variable to consider, especially in the context of diet-induced obesity during pregnancy. Previous work had demonstrated female C57BL/6 mice to be less susceptible to develop obesity and associated metabolic syndrome than males (Grove et al., 2010; Pettersson et al., 2012). Grove et al. (2010) found following a 12-week high fat diet regimen, females gained less weight than males; and following microarray analyses of adipose tissue depots between sexes, male adipose tissue exhibited a far more pronounced expression of inflammatory genes opposed to females. Their results being indicative of an inherent distinction between male and female adipose tissue, irrespective of diet. Pettersson et al. (2012) in contrast, found male and female mice to be similar in weight following a 14-week high fat diet regimen. However, further analyses of adipose tissue inflammation revealed notable sex differences (Pettersson et al., 2012). Male mice developed hyperinsulinemia and low-grade, systemic inflammation by means of an increased inflammatory macrophage population in intra-abdominal adipose depots. Whereas female mice did not display hyperinsulinemia or systemic inflammation, but instead displayed an increased anti-inflammatory T_reg_ cell population in adipose tissue in response to weight gain. These sex-dependent differences in response to high-fat diet exposure point to an interesting inflammatory mechanism that could be responsible for why female C57BL/6 mice appear (largely) resistant to obesity and ensuing metabolic syndrome. Since these studies, multiple groups have successfully modeled diet-induced obesity in female mice and further investigated potential immuno-metabolic mechanisms that play a role in resistance to metabolic changes; typically using approximately 45% kcal or a 60% kcal of fat diets (Samuelsson et al., 2008; Bruce et al., 2009; Ashino et al., 2012; Ge et al., 2014; Sasson et al., 2015; Singer et al., 2015; Bruder-Nascimento et al., 2017; Chang et al., 2019; Suarez-Trujillo et al., 2019; Fernandes et al., 2021; Losol et al., 2021; Zhang et al., 2021).

As the “Western” diet continues to take hold across the western world, there will be an ever-increasing proportion of high-risk pregnancies that will be susceptible to MIA and increased risk of NDDs due to maternal obesity. Therefore, it is crucial to begin incorporating this maternal state into future MIA animal investigations in conjunction with other traditional stressors (i.e., Poly I:C and LPS); while controlling for the effects of obesity-induced metabolic syndrome. Not only does this improve the credence of the MIA model but also its translational status. Fortunately, the diet-induced obesity model is very well-established in various rodent strains, ages, and developmental windows that the extension of such into a novel MIA study would not pose much of a challenge in terms of experimental design. With the above variables discussed in mind, the implications of modeling maternal obesity in the MIA space would provide valuable insight regarding the mechanisms driving fetal programming and subsequent neurodevelopment. However, the following experimental considerations should be addressed: (1) *Is the laboratory high-fat diet composition translationally relevant and optimal for use throughout pregnancy?* (2) *If using mice, is the strain being used for the study characterized as obesity-prone or resistant?* (3) *Are obesity-induced metabolic changes able to be monitored and/or controlled for during the study?*

### 2.5 Laboratory diet and food sterilization

Here, we shift the discussion from various inducers of maternal stress and subsequent inflammation to the importance of the maternal diet and modes of food sterilization. The composition of laboratory rodent diet is a variable that most research groups rarely consider or report on, unless conducting a diet study. But arguably, the choice of diet when designing an MIA animal study holds just as much weight as immunogen selection. We will discuss some key features that make up two of the most widely used laboratory rodent diets: grain-based (GB) and purified diets; and highlight their respective advantages and disadvantages. We will also discuss how phytoestrogen content in GB diets can influence experimental results and the implications of exposure during fetal development. Lastly, we will cover how sterilization processes can influence resulting nutrient composition and potentially, pregnancy viability.

Grain-based diets are cereal based and contain various combinations of natural ingredients such as soybean meal, ground corn, fish meal, wheat middlings, animal by-products, and high levels of insoluble and soluble fiber (Pellizzon and Ricci, 2020; Weiskirchen et al., 2020). There are advantages to using GB diets; they offer sufficient levels of essential nutrients for promoting growth and reproduction, animal colony maintenance and are relatively inexpensive (Pellizzon and Ricci, 2018). However, they tend to be more variable in nutrient content due to the diversity of ingredients involved, resulting in significant batch to batch variation. This source of variation is addressed by manufacturers through numerous quality control measures and altering the amounts of ingredients to ensure more nutrient consistency between batches (Pellizzon and Ricci, 2020). Unfortunately, these alterations to ingredient and nutrient composition are not always fully disclosed by manufacturers and is typically “closed” or proprietary information (Pellizzon and Ricci, 2018), limiting researchers’ ability to fully report the composition of their GB diet of choice in their studies. Therefore, if using a GB laboratory diet for MIA experiments, it is strongly recommended to employ a manufacturer that has an “open” formula (Barnard et al., 2009) or willing to disclose nutrient and ingredient composition upon request. This will allow for further transparency between both researchers and laboratory diet manufacturers, in addition to improving the reproducibility of the study. Importantly, GB diets not only present nutrient and ingredient variation, but also non-nutrients and contaminants. These include compounds such as phytoestrogens (Jensen and Ritskes-Hoitinga, 2007), heavy metals (i.e., arsenic) (Kozul et al., 2008) and synthetic contaminants, the levels of which vary depending on the manufacturer (Mesnage et al., 2015; Pellizzon and Ricci, 2020). While all of these factors present significant cause for concern about how they affect experimental results, what is probably most relevant in the context of MIA studies are the effects of phytoestrogen content.

Phytoestrogens are plant derived estrogenic compounds that mimic the structure and, in some instances, function of mammalian steroidal estrogens (Jensen and Ritskes-Hoitinga, 2007). There are three main categories of phytoestrogens that are relevant to both human and animal health: (1) isoflavones: derived from soybeans, (2) lignans: derived from large quantities of flax seeds, and (3) coumestrol: derived from alfalfa sprouts (Odum et al., 2001; Lephart et al., 2002). Many GB diets consist of either soybean and/or alfalfa meal as a source of protein, inadvertently leading to high levels of either isoflavones or coumestrol in animal feed. As soy is typically the main constituent of many GB laboratory diets, we will discuss isoflavones here in more detail. Isoflavones possess a striking resemblance to the 17-β-estradiol steroid hormone, allowing them to exert variable effects via binding to estrogen receptors, ERα and ERβ in humans and rodents (Lephart et al., 2002; Dixon, 2004; Jensen and Ritskes-Hoitinga, 2007). These variable effects are believed to be due to their ability to elicit both estrogenic and antioestrogenic effects and classifies them as “Selective Estrogen Receptor Modulators” (Cornwell et al., 2004; Jensen and Ritskes-Hoitinga, 2007). The two primary isoflavones in soybeans are genistein and daidzein in the form of glycosides (Messina et al., 2004). These isoflavones have been deemed the most potent in terms of estrogenic activity (Glazier and Bowman, 2001; Lephart et al., 2002; Jensen and Ritskes-Hoitinga, 2007) and as a result of soy being the principal source of protein in many laboratory diets, sustained, high steady-state isoflavone concentrations in the serum of rodents have been observed (ranging > 800 ng/mL) (Brown and Setchell, 2001; Thigpen et al., 2004; Jensen and Ritskes-Hoitinga, 2007). In addition to that, transplacental transfer of both genistein and daidzein has been demonstrated in studies done in rats (Doerge et al., 2001; Scott Weber et al., 2001; Degen et al., 2002), showing biologically relevant concentrations present in both the brain and serum of the offspring. Furthermore, genistein exposure pre- and postnatally has been reported to induce both reproductive (Jefferson et al., 2007; Cimafranca et al., 2010) and behavioral impairments (Wisniewski et al., 2005; Rodriguez-Gomez et al., 2014) such as decreased aggression, increased defensive behaviors and demasculinization in male mice. Additional studies have demonstrated these compounds to have profound effects on many aspects of sexual development as well; including changes to the timing of onset and indicators of puberty, impaired estrus cycling, ovarian function and altered hypothalamus and pituitary function (Odum et al., 2001; Thigpen et al., 2003; Kim and Park, 2012). Taken together, these studies emphasize the significance of phytoestrogen exposure on rodent sexual and behavioral development and demonstrate the potential of these dietary compounds interfering with critical periods of both prenatal and neonatal development. That being said, if selecting a maternal GB laboratory diet for an MIA study, research groups should attempt to address the following points: (1) *Is there a GB-alternative that is free of soy meal or alfalfa?* (2) *Can the phytoestrogen concentration be disclosed by the manufacturer? If not, are there resources available in the laboratory to quantify these levels?* (3) *Are the endpoints of the study sensitive to estrogen and/or antioestrogenic effects?*

Purified rodent diets on the other hand, are composed of highly refined ingredients and provide standardized, balanced compositions of nutrients. These compositions typically include corn starch as a source of carbohydrate, soybean oil as a source of fat, casein for protein, and cellulose for fiber (Pellizzon and Ricci, 2020; Weiskirchen et al., 2020). Since levels of both micro- and macronutrients are well defined for purified diets, nutrient variability and contamination between batches are small and individual ingredients can be readily manipulated to fit the specifics of a study. Two widely used purified diets today are the AIN-76 and AIN-93 series, which were devised by two separate American Institute of Nutrition committees in 1976 and 1993, respectively, to establish general nutrition and dietary guidelines for laboratory rodent studies (Reeves et al., 1993). Since their initial launch, subsequent alterations have been made to better support rodent growth and development and these typically serve as control diets for many animal studies. While these diets provide researchers a “cleaner” formula for maintaining consistency across studies, it is important to note that they may inextricably lead to an altered rodent phenotype compared to cohorts consuming a GB diet. The refined nature of the purified diet, presence of sucrose, relatively low amounts of total fiber and lack of fermentable fiber (cellulose is not fermentable by most gut microbiota), can alter the intestinal tract and contribute toward metabolic disease (Wise and Gilburt, 1980; Pellizzon and Ricci, 2018, 2020). Notwithstanding, modifications can easily be made to purified diets to add more total and soluble fiber sources or remove and/or substitute sucrose with corn starch for example. Therefore, the overall benefits of using a purified diet may outweigh potential metabolic risks. However, before committing to an AIN-series standard, it is worthwhile to examine if the desired experimental endpoints align with the dietary composition: (1) *Does the level of dietary refinement matter in the context of the MIA study?* (2) *Further, is the maternal gut microbiota a critical variable of the study? If so, does the desired AIN-series formulation require phenotyping prior to implementation in the study?*

Sterilizing laboratory diets is typically a necessity for many animal research facilities and common practice for specified-pathogen-free (SPF) animals. This is done to eliminate possible sources of microbial contamination that could lead to disease or infections among animal colonies and potential transmission within the larger animal facility. Due to sterilization processes being such common practice, research groups by and large have overlooked the significant influence they can have on resulting nutrient values and toxic compounds. These alterations can fundamentally affect various phenotypic parameters across generations of laboratory rodent models. Accordingly, we will discuss the two predominant sterilization methods used: steam autoclaving and irradiation, and their effects on overall diet composition and quality, fetal development, and breeding for MIA experimental design.

Autoclaving involves heat sterilization through a series of fluctuations in temperature and pressure. This method is rather inexpensive and effectively rids the feed of any microorganisms, viruses, and bacterial spores. There are no fixed standard autoclaving parameters for sterilizing animal feed but, most institutions use either 121°C or 135°C between 20 and 30 min (Ford, 1977; Twaddle et al., 2004; Weiskirchen et al., 2020), with one study suggesting the recommended sterilization temperature for SPF rodents be 121°C for 20 min (Tus̈nio et al., 2014). While autoclaving is certainly effective, it can introduce some negative effects on diet quality. There are reports of significant decreases in fat soluble vitamins A and D, and water soluble B1 (thiamine), alterations in isoflavone concentration, protein quality and bioavailability − as the amino acids lysine and cysteine are considered heat-sensitive (Collins et al., 1992; Tus̈nio et al., 2014; Kurtz et al., 2018). Therefore, manufacturers fortify their diets with additional vitamins and proteins to compensate to make diet formulations more suitable for autoclaving. A notable observation from multiple groups is that following autoclave sterilization, there are increases in food pellet hardness (Ford, 1977; Kurtz et al., 2018; Weiskirchen et al., 2020). This introduces expected changes in the way the animals can effectively consume their feed and resulting wastage. In a study done in DBA/1 and LACA strains of mice, it was observed that mice fed a “commercially” available autoclaved pelleted diet reduced overall food wastage but also true consumption of the diet (Ford, 1977).

Besides compromising nutritional value and altering a diets’ physical properties, autoclaving is also known to result in the production of acrylamide − an industrially produced, reactive crystalline solid used to synthesize polyacrylamide (Friedman, 2003). This compound can be found in cosmetics, paper, and textile industries, in addition to the laboratory in the form of polyacrylamide gel for the separation of proteins by electrophoresis (Friedman, 2003). It is also considered to be a potent neurotoxin, genotoxin, and multiorgan carcinogen in laboratory animals and a likely carcinogen in humans (Rice, 2005; Kurtz et al., 2018). Moreover, a study done by Kurtz et al. (2018) demonstrated levels of acrylamide produced from autoclaving standard rodent feed directly corresponded to increasing sterilization temperatures − with levels being comparable to the estimated dietary intake in humans from heated foods. Dietary exposure to acrylamide in humans is formed as a by-product of cooking everyday foods when reducing sugars (i.e., fructose and glucose) react with the amino acid asparagine during a chemical reaction called the Maillard reaction (Virk-Baker et al., 2014). Products of this reaction are what causes food to brown and are responsible for much of the taste from baking, frying or roasting (Mottram et al., 2002). Since its discovery in the human diet and autoclaved rodent feed, many have studied the physical and chemical properties of acrylamide and the associated risks of exposure to it extensively. It is becoming increasingly more important to consider the parameters used for routinely autoclaving animal feed institutionally and how this potential toxicity may affect the interpretation of resulting data. Therefore, research groups should consider the following when it comes to autoclaving laboratory feed: (1) *Are the institutional autoclaving procedures appropriate for the study?* (2) *Can the laboratory diet manufacturer share details regarding the degree to which nutritional value is altered following autoclaving? Palatability of the diet? Potential polyacrylamide levels?* (3) *If the diet is not suitable for autoclaving, are there reasonable alternatives that will preserve the formulation and not affect downstream analyses?*

Gamma-irradiation on the other hand, involves the use of γ-rays as a means to destroy microorganisms present in animal feed and reduces possible diet spoilage. This process is a suitable sterilization alternative to autoclaving and appropriate for animals housed under SPF conditions or immunocompromised. The irradiation dosage is measured in kiloGrays (kGy), with one Gray (Gy) referring to the absorption of one joule (J) of radiation energy per kilogram of material (1 Gy = 1 J/kg) (Weiskirchen et al., 2020). Standard dosages used range from 20 to 30 kGy and higher doses between 40 and 50 kGy are typically used for germ-free animals where food sterility is even more critical (Caulfield et al., 2008). The preferred dosage used by the manufacturer’s irradiation facility is typically disclosed upon the investigators’ request and depending on the specific requirements of the study, the dose can be modified. The irradiation process itself, requires a controlled concentration of γ-rays to be exposed to the animal feed from a radioactive source; which in commercial radiation facilities is usually cobalt-60 (Caulfield et al., 2008; Weiskirchen et al., 2020). A defining feature of γ-radiation is its impressive capability to penetrate material, making it a highly effective method for destroying any lingering pathogens that may be present in animal feed and ensuring sterility (Kilcast, 1994; Caulfield et al., 2008). However, the way in which ionizing radiation works involves the production of highly reactive free radicals. These molecules react with the DNA of living microorganisms causing cell death (Kilcast, 1994). These free radical by-products have implications not only for the integrity of the animal diet, but possibly the overall health of the animal as well. It has been well-established that γ-irradiation of animal feed leads to the reduction of vitamin content, alters vitamin stability, and compromises fatty acid composition. An interesting study examined the effects of “standard” (28.9−34.3 kGy) and “high-end” (38.4−48.7 kGy) dosages of γ-irradiation on protein, fat, and carbohydrate present in commercially available cat, canine, and rodent feeds (Caulfield et al., 2008). They observed that γ-irradiation increases peroxide content across all diets. While significant reductions in vitamin A following both dosages were observed solely in the cat diet, only the high dosage reduced vitamin A in the rodent diet, and no changes to vitamin A levels were observed in the canine feed. An earlier study examined the effects of γ-irradiation on vitamin content in laboratory feed for germ-free chickens, SPF cats and guinea pigs (Coates et al., 1969). Coates et al. (1969) discovered there to be significant reductions in vitamins C, B1, E, and A using an irradiation dosage between 20 and 50 kGy. Fatty acid content is another important aspect of nutrition for any animal diet and proven to be quite challenging to preserve during the sterilization process – whether it be autoclaving or γ-irradiation. Ionizing radiation, as alluded to previously, generates many free radicals that work to eliminate various microorganisms. But in the context of fat preservation, this method is arguably, less than ideal. A study investigating the effects of ionizing radiation on fatty acid composition and lipid peroxidation products, observed a striking destruction (98%) of polyunsaturated fatty acids that was associated with an increase in lipid peroxide formation following doses of 2−10 kGy (Hammer and Wills, 1979). High dosages of γ-irradiation can result in the formation of lipid peroxides, the extent of which depends on several factors including, lipid composition, irradiation dosage, temperature, oxygen, and antioxidants of course (Hammer and Wills, 1979). Notably, unsaturated fats are most vulnerable for undergoing lipid peroxidation. The ensuing lipid peroxides have been demonstrated to result in the formation of insoluble plaques, that can build up within blood vessels (Tritsch, 2000; Harrell et al., 2018). Lipid peroxides can also be extremely reactive and depending on concentration, can disrupt cell signaling processes, cause protein and DNA damage, and cytotoxicity (Ramana et al., 2019). While the introduction of γ-irradiation may be considered indirect, as the γ-rays are only directly exposed to the feed and not to the animal, the oxidative by-products, and any additional residual degradation products, could have indirect effects on the animal’s metabolism and fertility. Maternal exposure of such could in turn, result in persistent changes to the epigenome that could be transmitted to generations of offspring, and lead to inaccurate interpretations of MIA data if not accounted for. Accordingly, some points for groups to consider regarding γ-irradiation: (1) *Is the maternal diet of choice sensitive to the effects of γ-irradiation sterilization? If so, at what dosage?* (2) *If the study opts for a high-fat maternal diet – is γ-irradiation necessary?* (3) *Can the production of lipid peroxides be measured before administering the diet to the animals?*

### 2.6 Parental genetics and impact on the immune system

It is well-known that there is immunological variation between different laboratory strains of rodents. In fact, one of the most widely recognized differences in immunological responses lies within the commonly used C57BL/6 and BALB/c strains. C57BL/6 mice demonstrate a subset of helper T (Th) cells termed, Th-1-predominant response to pathogens, while BALB/c strains typically exhibit a Th-2-predominant response (Sellers et al., 2012). In brief, Th-1 cells facilitate the elimination of intracellular pathogens, whereas Th-2 cells mediate responses to parasitic infections (Sellers et al., 2012). How each modality brings about these respective responses, involves a great deal of nuance outside the scope of this review, but many typically arrive at the dichotomous M1/M2 macrophage paradigm that has sparked some controversy over the years. Briefly, this dichotomy of a Th-1 vs. Th-2 response (and M1 vs. M2) is an oversimplification as it is becoming increasingly clear that helper T cell and macrophage responses exist on a continuum. Further discussion concerning the details of this paradigm can be found here: (Nahrendorf and Swirski, 2016; Ley, 2017). For the purposes of this review, essentially the Th-1-mediated immune response generates IFN-γ which predominantly activates macrophages; the Th-2-mediated in contrast, generates IL-4, IL-5, and IL-10, which largely inhibits macrophage activation and stimulates antibody production instead (Mills et al., 2000). These responses are part of a larger spectrum of immune processes, in line with differing states of activation and differentiation of immune cells, including macrophages and Th cells (Nahrendorf and Swirski, 2016). The important distinguishing factor between the states is not one of activated vs. an inactivated status, but their propensity to express different metabolic programs. Sellers et al. (2012) found M1 macrophages to mainly facilitate the Th-1 response and generate higher levels of nitric oxide following IFN or LPS exposure as opposed to M2 macrophages, who mainly facilitate the Th-2 response and generate polyamines (Mills et al., 2000). Therefore, each host of immune responses and associated macrophage activity undoubtedly affects the outcomes of rodent studies involving infection and immunity.

Mouse models of asthma for instance, have been examined in at least nine different strains (Whitehead et al., 2003) and results from these experiments illustrate the significance of genetics on immune reactivity. For example, a study done by Schwartzer et al. (2017) investigated maternal allergy and asthma during pregnancy as a model of MIA in mice. They used two immunologically distinct mouse strains (C57BL/6 and FVB/Ant) that present with differing allergen-induced asthmatic responses. As described by Schwartzer et al. (2017), the FVB/Ant strain displays a more heightened Th-2 mediated allergic-asthma response following allergic asthma induction than C57BL/6 mice and similar to what has been described in Balb/c mice (Gueders et al., 2009). In line with this, the C57BL/6 strain, displays a dampened Th-2-response (i.e., Th-1-dominant) in an ovalbumin-induced allergy model (Zhu and Gilmour, 2009). In brief, the authors demonstrated strain-dependent behavioral changes in offspring born from maternal allergic asthma (MAA) dams. The C57BL/6 MAA offspring demonstrated reduced social interactions with a strain-matched control mouse, while FVB MAA offspring demonstrated more social engagement. This data is also consistent with Schwartzer et al.’s (2013) earlier work highlighting strain-specific interactions following poly I:C-induced MIA between C57BL/6 and BTBR T ^+^ tf/J mice. Overall, strain-specific immunity has the potential to affect downstream analyses and underscores the importance of strain selection when planning and reporting an MIA investigation. What is perhaps even more interesting, is that the above reports are the sole studies to our knowledge, that have raised the question of whether a Th-2 mediated maternal immune response presents the same risk of neurodevelopmental disease or associated pathology as a Th-1 mediated response. This question opens the door for further comparative MIA studies between several commonly used rodent strains (i.e., C57BL/6/Balb/c) and could yield compelling results surrounding Th-1 and Th-2 mediated maternal immunity and offspring brain pathology and behavior. Both traditionally used immunogens, LPS and poly I:C, drive a Th-1 mediated immune response (McAleer and Vella, 2010), which will affect BALB/c and C57BL/6 mice differently and subsequently, their offspring.

Investigators should also be aware of which genetic strains are more resistant and susceptible to particular pathogenic stimuli. Inbred laboratory mice strains display immune response patterns that continue to depart from previous generations, as a consequence of numerous mutations and polymorphisms (Sellers et al., 2012). If one is uninformed about some of the defining characteristics of a specific strains’ immune response, this can undermine accurate data interpretation; especially when unraveling how the maternal immune response influences various physiological responses in the offspring. SJL/J mice for instance, are described by The Jackson Laboratory − as being immunocompetent but displaying elevated levels of circulating T cells – a variable of the adaptive immune system that should be taken into consideration when performing immunological analyses (The Jackson Laboratory^1^). To provide another example, we turn to *Tlr4* mutant mice. To reiterate what has been previously described, TLR-4 is the primary receptor for recognizing LPS and is used in a substantial number of MIA studies. The C3H/HeJ mouse strain (and associated strains) possess a point mutation in the *Tlr4* gene that renders the TLR4 receptor unresponsive to LPS and consequently, these mice become more susceptible to gram-negative infections such as, *Escherichia coli* (Suhs et al., 2011). It has also been documented that C57BL/6 mice are inherently more susceptible to infection from *Mycobacterium paratuberculosis* as compared to C3H/HeN mice (Veazey et al., 1995). This is due to C57BL/6 mice carrying a susceptibility allele for the *Bcg (Nramp1)* gene that controls natural resistance to the bacterium (Buu et al., 2000). The role of the complement system is another important player of innate immunity and a system posited to be “primed” by MIA during fetal development (Knuesel et al., 2014). There are various mouse strains (A/J, AKR/J, DBA/2, DBA/1, FVB/NJ, and SWR) that possess a loss-of-function mutation in complement component 5 (C5), due to a frame shift mutation in their *hemolytic complement* (*Hc)* gene (Wetsel et al., 1990; Sellers et al., 2012). C5 is an important part of a larger family of complement proteins that broadly facilitate phagocytosis, inflammation and cytolytic processes in response to pathogens (Wetsel et al., 1990; Chen et al., 2010). On the whole, strains that possess this mutation are more susceptible to infections from *Bacillus anthracis, Aspergillus fumigatus*, and *Candida albicans* compared to the C57BL/6 strain which possess intact C5 function (Harvill et al., 2005; Svirshchevskaya et al., 2009; Radovanovic et al., 2011).

Against this background, perhaps a simple declaration of the hosts predominant innate immune response will become common practice and spark more meaningful discussion thereafter. However, there has been exploration of gene-environment interactions in MIA models that have tip-toed around the idea of genetic background-specific effects and susceptibility to disease. This has largely been done in the context of introducing human susceptibility-genes known to predispose an individual to schizophrenia or autism-related risk in addition to maternal poly I:C exposure (Abazyan et al., 2010; Vuillermot et al., 2012; Lipina et al., 2013; Wu et al., 2015). The activation of the maternal immune system is driven by a multitude of factors and with that, it can be presumed that a breadth of immune responses take place during pregnancy that ultimately render a child to be at higher risk of disease incidence. Therefore, if the goal of modeling MIA in animals is the ability to confidently translate observations to the human population, it would be advised to bear in mind the following: (1) *Ensure the implementation of Th-1 and Th-2 specific stimuli are incorporated into the appropriate model* (2) *Is the genetic background of the model predisposed to a Th-1 or Th-2 dominant response?*(3) *Have the background genetics dictating host immunity been well-characterized?*

## 3 Offspring effects of MIA and analyses

Here, we shift the discussion from variables to consider when designing a maternal focused MIA experiment, to further experimental considerations when analyzing MIA offspring. Measures of prenatal stress due to MIA should not be confounded with factors that could affect the perinatal environment, therefore skewing any offspring behavioral or biochemical data. In other words, the perinatal environment is just as important to control as the environment *in utero*.

### 3.1 Neonatal handling and cage-change frequency

When disclosing offspring analyses, most MIA reports typically include the date of weaning and/or sexing, the timing of subsequent behavior testing and if both sexes were included in the following analysis. However, details regarding the neonatal period prior to weaning are often rarely reported. Yet, the neonatal period is considered just as sensitive a time for neurodevelopment as the prenatal period and can present an important source of biological variation if not accounted for. The primary aim of many MIA investigations is to uncover deterministic mechanisms specific to the *in utero* environment that could be responsible for biochemical and behavioral deficits akin to NDDs. Nonetheless, many investigations tend to place the most emphasis on the effects of the prenatal environment instead of the neonatal period as well.

Most of what we have come to understand about the effects of neonatal stress, early rearing environments and handling effects on the developing nervous system has largely come from studies in rats (Levine, 1956; Levine et al., 1967; Wakshlak and Marta, 1990; Smythe et al., 1996; Vallée et al., 1997). This area of study surfaced substantially from early work by a distinguished psychobiologist, Seymour Levine who was intrigued by the idea that early life experience had lasting effects on behavior and personality development (Stern et al., 2010). Levine et al. (1967) have been credited as the first to develop a rodent model of early-life trauma and to demonstrate the enduring effects on neurodevelopment (Levine, 1956, 2002). An exceptional commentary on the contributions of his work can be found here: (Stern et al., 2010).

The first 2 weeks of life in rodents involve extensive neuronal plasticity and include the orchestration of the following processes: synaptogenesis, dendritic outgrowth and formation of the neural circuitry (Parfitt et al., 2004). Attention to the frequency of cage changes and neonatal handling prior to weaning has been recognized to have lasting effects on offspring behaviors and response to stress; and in a sex-specific manner as well. Neonatal handling refers to the separation of pups from the dam for a short period daily (the timing of which typically ranges between 3 and 15 min), during the neonatal period prior to weaning (Akatsu et al., 2015). Protective benefits have been overwhelmingly observed amongst offspring subject to this paradigm, where neonatal handled rats have been described to exhibit an overall better-modulated or adaptable response to stress following several endocrinological and behavioral testing (Raineki et al., 2014). Findings supporting this notion span from numerous observations that demonstrate increased glucocorticoid receptor density and receptor binding in the hippocampus (Meaney and Aitken, 1985; Meaney et al., 1985a,b, 1988, 1989; vishai-Eliner et al., 2001) to reduced stress hormone secretion and a speedier return to baseline levels following various stress-inducing stimuli in handled vs. non-handled animals (Levine et al., 1967; Hess et al., 1969; Weinberg and Levine, 1977; Plotsky and Meaney, 1993; Meerlo et al., 1999; Liu et al., 2000). An excellent review discussing the extensive effects of neonatal handling in further detail can be found here: (Raineki et al., 2014). In addition to the beneficial effects appeared to be inherent to neonatal handling, few studies have found this convention to rescue anxiety-like behavior and even hippocampal neurogenesis following exposure to various prenatal-stressors during pregnancy (Wakshlak and Marta, 1990; Lemaire et al., 2006; Bogoch et al., 2007). This collection of data raises questions regarding the relevance of the neonatal period in the context of an MIA investigation. While those carrying out MIA studies are not actively conducting neonatal handling experiments in and of itself, the frequency of cage changes prior to weaning can potentially have influence on downstream analyses of the offspring. This source of variation is in line with differences observed between two caging systems (i.e., open cage and individually ventilated cage systems) on the outcomes of MIA in mice (Mueller et al., 2018).

The frequency of cage-changes varies across laboratory animal facilities and while it remains an essential practice to ensure good health and wellbeing to both the animals being housed and humans working with those animals, it can also prove to be disruptive to mice. Rodents are incredibly scent-driven in nature, where a “stress-free” microenvironment for them equates to a pheromone- and biological waste-laden cage that humans would often constitute as “dirty.” An investigation prolonged the cage-changing period to 17 days and analyzed the effects on health and welfare on ICR female mice (Rosenbaum et al., 2009). The authors note this strain of mice were selected due to their inherently larger size and with a capacity of 5 mice per cage to model a microenvironment that would serve as a more extreme example of a “dirty” cage. Overall, the 17-day cage-change interval did not give rise to any detrimental effects on the microenvironment within the cages (i.e., ammonia levels, temperature, and humidity) and wellbeing of the mice (i.e., body mass, coat condition, behavior, fecal corticosterone, and pulmonary cytology). Another study tested 7-, 14-, and 21-day cage-change frequencies on C57BL/6J breeding performance, weanling weight and growth, plasma corticosterone levels, immune function, and histology on selected tissues (Reeb-Whitaker et al., 2001). They observed reducing cage-change frequencies to every 14 or 21 days did not present any detrimental effects on the above parameters. While the number of pups born remained unaffected, they did find pup mortality to be highest when cages were changed every 7 days; suggesting a cage-change frequency once every 14 or 21 days may be more beneficial to ensure pup survival. Discussions revolving around the improvement of animal welfare by reducing the frequency of cage changes have been relatively scarce in recent years (Reed et al., 2008) and with the number of MIA studies in rodents on the rise, warrants further dialog. Nonetheless, preliminary data points to no adverse effects on the health of the mice.

Thus, cage changes are yet another variable that needs to be considered. Many laboratory animal facilities carry out cage-changes within a 3-to-7-day range (Rosenbaum et al., 2009) and therefore presenting this modification to ones’ resident animal ethics or welfare committee may present challenges. In the case of an MIA investigation where parameters of stress must be tightly controlled, a modification in this case may be justified. In addition, it may elicit more robust behavioral and biochemical data in MIA offspring if they are left undisturbed with the mother prior to weaning. In the context of neonatal handling and cage-change frequency, investigators should consider the following points: (1) *Can neonatal-handling be monitored during the study?* (2) *Can cage-change frequency be reduced during the neonatal and/or weanling period?* (3) *Is the context of the MIA study sensitive to these parameters?*

### 3.2 Sex differences

A strong sex bias is arguably at the heart of several NDDs, such as ASD, ADHD, and SCZ (May et al., 2019; Merikangas and Almasy, 2020). Yet, the precise mechanisms responsible for such remains unclear and many animal studies predominantly analyze males. ASD is a prevailing example of a disease that presents with a striking male bias, where the relative male: female ratio is estimated to be 4:1 (Werling and Geschwind, 2013; Lord et al., 2020). ADHD also appears to possess a similar bias, with increased prevalence amongst males with an estimated male: female ratio of 3:1 (Hanamsagar and Bilbo, 2016). The provocative “female protective effect” hypothesis has been proposed which stems from human genetic analyses suggesting females may require a higher mutational burden or etiologic load to manifest autism associated impairments (Robinson et al., 2013; Jacquemont et al., 2014). While in the context of male vulnerability to NDDs, the early “extreme male brain” hypothesis was proposed which posits autism to arise from hypermasculinization of the brain and driven in large part by fetal testosterone exposure (Baron-Cohen, 2002; Auyeung et al., 2006, 2009). However, these examples are only two of many emerging hypotheses that attempt to unpack the extremely complex sex bias that lies within the manifestation of many NDDs, and with limited empirical support, warranting further study. Unfortunately, a large part of MIA-animal data either do not include “sex” as a biological variable in downstream analyses or do not investigate the many nuances of sex-specificity which is extremely relevant in the etiology of several NDDs. In addition, male and female analyses are often pooled together rather than analyzed independently. An exceptional commentary on the profound sex disparity amongst MIA animal models and basic neuroscience research as a whole, can be found elsewhere (Coiro and Pollak, 2019).

Sexual dimorphism is also often observed in the context of the immune system as a whole and may lend additional insight as to why males fare worse than females in the development of NDDs. Overall, adult females produce a more robust innate and adaptive immune response than males (Klein and Flanagan, 2016). This rapid clearance of pathogens is believed to be due in part, to higher circulating levels of immunoglobulins IgM and IgG in females (Gal-Oz et al., 2019). However, this response also comes with greater risk of SCI and subsequent autoimmune disease. These observations have been confirmed at the transcriptomic level in mice, where females were observed to possess higher baseline levels of activated T cells and immunoglobulins (Gal-Oz et al., 2019). Single-cell sequencing results from the same study also found higher expression of interferon and complement-related genes in females exclusively in macrophages derived from the peritoneal cavity, spleen, and microglia in comparison with males. These data are indicative of an enhanced ability in females to fend off various infectious diseases, but at the cost of a higher susceptibility to autoimmune diseases. Of which tend to affect women during periods of exceptional stress or hormonal changes such as, pregnancy (Angum et al., 2020). Males on the other hand, are more vulnerable to pathogenic infections and often with increased severity, beginning as early as birth and lasting through adulthood (Klein and Flanagan, 2016; Chamekh and Casimir, 2019; Gal-Oz et al., 2019; Jaillon et al., 2019). Male newborns experience more cases of sepsis and meningitis than females; then beginning in early childhood, they become more at risk for developing various respiratory and viral infections (Libert et al., 2010; Snell and Turner, 2018). These observations lead to the proposition that a large part of what contributes to this dimorphism in innate immunity lies within the X chromosome.

While the fine details of what constitutes a male and female embryo is outside the scope of this review, here we will provide a brief context that will be important for understanding how the X chromosome confers an immunological advantage. Essentially, the X chromosome is comprised of several genes that are either directly or indirectly involved in the regulation of the immune response, many of which are polymorphic (Spolarics, 2007). Females carry two X chromosomes, while males only carry one. Therefore, females inherently carry two copies of X-linked immunomodulatory genes, whereas males only carry one copy of these genes. If females were to express double the amount of these genes resulting in double the dosage of proteins in all cells, the physiological effects of such would be incredibly detrimental. Therefore, to circumvent this potential damage, an evolutionarily conserved process termed X-chromosome inactivation emerged that randomly silences one of the X chromosomes during the early stages of female embryogenesis (Libert et al., 2010; Sahakyan et al., 2017). Where the advantage lies for females is that this process results in cellular mosaicism (i.e., half of cells express genes derived from the maternal X chromosome, the other half express genes derived from the paternal X chromosome), which confers an additional layer of genetic diversity that confers protection from both deleterious X-linked chromosome mutations and new immune challenges (Spolarics, 2007; Libert et al., 2010). Males in contrast, bear the unfortunate risk of manifesting most recessive X-linked mutations, that explains the increased prevalence and severity of several X-linked primary immunodeficiencies amongst males (Libert et al., 2010). However, as mentioned previously, females do experience a predominant predisposition to several autoimmune diseases some of which have been posited to be at the expense of skewed X chromosome inactivation patterns over a woman’s lifetime (Ozcelik, 2008; Selmi, 2008). Excellent reviews further delineating the effects of sex chromosomes on innate immunity and inflammation can be found elsewhere: (Spolarics, 2007; Libert et al., 2010). Taken together, sex chromosomes are critical players in both the etiology and pathogenesis of several diseases and can perhaps help us untangle some of the complexity that lies within the ever-so evident sex biases observed in a host of NDDs.

### 3.3 Male vulnerability and female resilience

Of the few animal studies that have opted to analyze male and female offspring independently, a clear sexual dimorphism following MIA exposure appears to corroborate with what is seen amongst human NDD patients. A notable MIA mouse study examining autism-associated behaviors in both male and female offspring born to mothers exposed to either LPS or Poly I:C during mid-gestation, found significant interactions between “treatment” and “sex” (Xuan and Hampson, 2014). Specifically, male offspring from LPS or Poly I:C treated dams demonstrated increased repetitive behaviors via the marble burying test, while female LPS and Poly I:C offspring did not exhibit any significant increases compared with control females. Male Poly I:C and LPS offspring appeared to show normal social behaviors with only LPS female offspring displaying modest social deficits. Another study observed males exhibited reduced social interactions and deficits in motor development and coordination deficits following prenatal Poly I:C exposure, whereas Poly I:C females did not exhibit changes in sociability and displayed minor developmental delays (Haida et al., 2019). These deficits in males also appeared to be reflected by reduced numbers of Purkinje cells in the cerebellum and reduced numbers of neurons in the motor cortex. A recent study harnessed single-cell RNA sequencing technology to better understand the molecular mechanisms underlying MIA following Poly I:C exposure and again, found sexually dimorphic effects between male and female offspring (Kalish et al., 2021). Kalish et al. (2021) observed marked sex-specificity in the context of proteostasis and the integrated stress response (ISR) – another relevant mechanism recently implicated in the etiology of NDDs gaining momentum (Lombardo et al., 2018; Advani and Ivanov, 2019; Chen et al., 2019; Zengeler and Lukens, 2021). In brief, Kalish et al. (2021) observed the activation of the ISR triggered by ER stress, to be at the core of social and repetitive behavioral deficits in male MIA offspring. Together with that, when the ISR pathway was genetically ablated, social, and repetitive behavioral deficits were rescued. Female MIA offspring on the other hand, did in fact exhibit transcriptional changes in response to MIA as well, but did not display the same degree of disruption to translational machinery as males. These findings suggest females exert better translational control under prenatal stress which may also feedback into the notion that a higher mutational burden is required for females to manifest several NDD-associated impairments.

A related perspective on male vs. female vulnerabilities to disease onset following prenatal MIA exposure is illustrated by Braun et al. (2019). Following a battery of analyses focusing on the relative abundance of cytokines in the fetal brain and placenta, adult growth and behavioral testing, their findings describe a unique set of developmental alterations between male and female offspring. In essence, while males still suffered more severe health outcomes in response to maternal inflammation (i.e., elevated placental inflammation, reduced placental growth, decreased social interactions, and impaired working memory); females were not entirely “protected” and presented with their own host of developmental consequences, including but not limited to: moderate placental inflammation, delayed adult growth, decreased exploration, and altered excitatory and inhibitory neuronal densities in the cortex. These data suggest that the male and female response to MIA is incredibly unique; and therefore, more experiments should incorporate both male and female offspring to gain a more complete understanding of what mediates differences in NDD incidence rates and severity. On that note, future studies should consider the following: (1) *Will offspring analyses of both sexes be included in the study? If so, is a rationale provided for pooling both sexes together and/or keeping them independent* (2) *Are there sex-specific variables that warrant disclosure or additional control?* (3) *Are any aims of the study oriented to address the sex bias present in many NDDs?*

## 4 Discussion

As the prevalence of NDDs continues to rise, a better understanding of the biological mechanisms underpinning these diseases can only be achieved with a reproducible MIA animal model. The MIA experimental system that the international scientific community has come to know serves as an incredibly powerful tool for both researchers and clinicians, for understanding the complex etiology behind NDDs. Therefore, it’s important to recognize the experimental parameters behind the model that support its validity and those that undermine it. Productive discussions and action regarding this have taken place over the years and more recently, an MIA model reporting checklist has been proposed (Kentner et al., 2019). The checklist includes several guidelines, such as the number of experimental and control groups, details regarding compounds and/or immunogens used, route of drug administration, species/strain/vendor, housing, and husbandry, experimental outcomes, and additional disclosures. Adherence to this checklist moving forward is critical for improving transparency and raising awareness of the many experimental variables that can hinder accurate data interpretation and the advancement of the MIA model. Since its’ launch, only few publications, to our knowledge, have included a reporting checklist as supplementary material (Kreitz et al., 2020; Mueller et al., 2021). However, it is expected to be implemented more frequently as the details regarding the rigor and reproducibility of the MIA model become even more transparent.

## 5 Conclusion

We have delved deep into the depths of MIA model experimental design and a host of extraneous variables that have the potential to act as confounds. Some of which have been raised in previous discussions, such as the variability surrounding the utility of Poly I:C and have made a lasting impact on the widespread use of the Poly I:C-based MIA animal model. While other environmental variables such as, cage change frequency, neonatal handling, and laboratory diet on the other hand, have been left out of the larger MIA model conversation. These variables can be described as “hidden variables” (Butler-Struben et al., 2022) and are ubiquitously present in all experiments. It is our hope that by drawing immediate attention to these additional aspects of experimental design, the integrity of subsequent animal MIA models will greatly benefit. With this model coming under increasing scrutiny, it is important to control for the most important parameters where possible and disclose valuable information to inform the larger scientific community moving forward. The MIA model is unique in that it is incredibly malleable and with that, its use has led to a diversity of discoveries that have clued in both preclinical and clinical researchers alike, on what causal mechanisms may be at work. Moving forward, appropriate disclosures and standardized experimental design will be paramount for driving two important clinical goals: (*1)* How can we accurately predict which pregnancies to be most susceptible to the neurodevelopmental risks of MIA on offspring; and (*2)* how can we successfully intercept and potentially reverse the consequences of this prenatal adversity for individuals afflicted with various NDDs.

## Author contributions

MB wrote the manuscript. All authors have made a substantial, direct, and intellectual contribution to the work, and approved it for publication.

## Footnotes

1
https://www.jax.org/strain/000686
